# System Description and First Application of an FPGA-Based Simultaneous Multi-Frequency Electrical Impedance Tomography

**DOI:** 10.3390/s16081158

**Published:** 2016-07-25

**Authors:** Susana Aguiar Santos, Anne Robens, Anna Boehm, Steffen Leonhardt, Daniel Teichmann

**Affiliations:** Philips Chair for Medical Information Technology, RWTH Aachen University, Pauwelsstrasse 20, Aachen 52074, Germany; anne.robens@rwth-aachen.de (A.R.); boehm@hia.rwth-aachen.de (A.B.); leonhardt@hia.rwth-aachen.de (S.L.); teichmann@hia.rwth-aachen.de (D.T.)

**Keywords:** electrical impedance tomography, multi-frequency, time-difference imaging, frequency-difference imaging, complex impedance, FPGA, bioimpedance spectroscopy

## Abstract

A new prototype of a multi-frequency electrical impedance tomography system is presented. The system uses a field-programmable gate array as a main controller and is configured to measure at different frequencies simultaneously through a composite waveform. Both real and imaginary components of the data are computed for each frequency and sent to the personal computer over an ethernet connection, where both time-difference imaging and frequency-difference imaging are reconstructed and visualized. The system has been tested for both time-difference and frequency-difference imaging for diverse sets of frequency pairs in a resistive/capacitive test unit and in self-experiments. To our knowledge, this is the first work that shows preliminary frequency-difference images of in-vivo experiments. Results of time-difference imaging were compared with simulation results and shown that the new prototype performs well at all frequencies in the tested range of 60 kHz–960 kHz. For frequency-difference images, further development of algorithms and an improved normalization process is required to correctly reconstruct and interpreted the resulting images.

## 1. Introduction

Electrical impedance tomography (EIT) is a method for imaging the impedance distribution of the human body in a cross-section of the thorax. It relies on the injection of small alternating currents and measurement of the resulting voltages at the thorax surface. EIT is a non-invasive bedside method, harmless to the patient, and is a real-time diagnostic tool [1,2,3]. Several EIT systems have been developed by various research groups worldwide [4,5,6,7,8]. Traditional EIT systems are based on single-frequency measurements and time-difference imaging. With time-difference imaging, fast impedance changes in the body, induced by pulmonary or cardiac functions, can be reliably monitored. However, small and slowly evolving impedance changes caused by, e.g., atelectasis or pulmonary edema, may lead to misleading interpretation, since an increase of impedance might be due to an increase in liquid content (pulmonary edema) or to a decrease of air volume (atelectasis) [9]. Furthermore, for quantification of the amount of atelectasis/edema, a reference measurement of the patient in a healthy condition is necessary but is, generally, not available.

It is known from bioimpedance spectroscopy that by measuring tissue impedance at different current frequencies, intracellular and extracellular cell compartments can be distinguished [10]. Multi-frequency EIT (mfEIT) is a technique that makes use of current injection at multiple frequencies, to take advantage of the frequency-dependance of the complex impedances of tissue. In these systems, although time-difference images at multiple frequencies have mainly been used [11,12,13], frequency-difference imaging is of increasing interest [14,15,16] as this may provide additional information on tissues and, therefore, extra diagnostic information; moreover, frequency-difference imaging does not need a reference in time [15]. Although these studies have been mostly performed for stroke imaging, lung imaging might also be a promising application area. Nevertheless, to the best of our knowledge, frequency-difference images have only been presented from measurements on static phantoms or simulations, whereas measurements on animals or humans have been presented with time-difference images only [15,16,17].

mfEIT systems require the control, generation, acquisition and signal-processing of high frequency signals. For that, multiple functions, switches, multipliers and other logic have to run at a very high speed in order to keep all the processes in parallel and in real time. Additionally, each frequency of the acquired data has to be processed for both real and imaginary components separately and be transferred to a personal computer (PC). Therefore, a main controller with a reconfigurable architecture and a parallel operation, such as a field-programmable gate array (FPGA), is appealing [17]. This enables the design of a programmable and flexible device that is ready for interoperability with other medical devices, such as an ECG.

Here, we present the hardware/software co-design of a serial FPGA-based EIT system, including the signal generation for current injection, voltage measurement and demodulation, control of the measurement method and data transfer. In contrast with the device in [17], which is a parallel system, our system is a serial one, resulting in a simpler design. Since it only incorporates one current source and one measurement channel, the system is much smaller and hence cheaper, and the exhausting calibrations between multiple independent current sources and measurements channels are not an issue. Furthermore, multiple sources produce also more noise, and the inaccuracies associated with the implementation of extra hardware may overlay its advantages [18]. Hence, a serial system was developed allowing multi-frequency operation by means of multi-sine waveform. The system was developed with the main focus in frequency-difference imaging and time-difference imaging for regional lung ventilation, but not restricted to it. We combined this system with high frame rates capability, so that in future investigations, it would be enough for perfusion monitoring, additionally to the regional ventilation monitoring.

Measurements are performed in a resistive/capacitive (static) test unit and during a self-measurement (in-vivo measurement). Time-difference images and frequency-different images are reconstructed and presented for both measurement sets.

## 2. System Design

**Figure 1:**
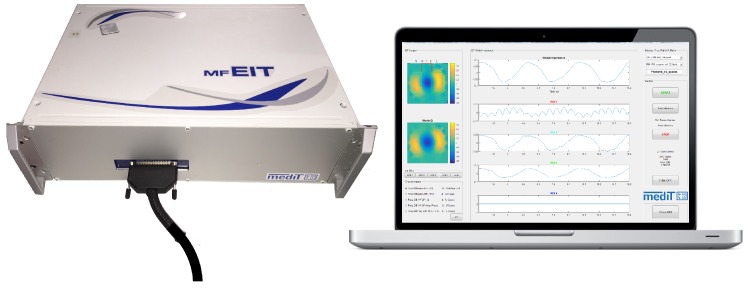
Prototype of a multi-frequency EIT system.

The mfEIT prototype is a 16-electrode serial system, with one current source and one measurement channel multiplexed through all electrodes; Figure 1 presents a prototype of the system.

**Figure 2:**
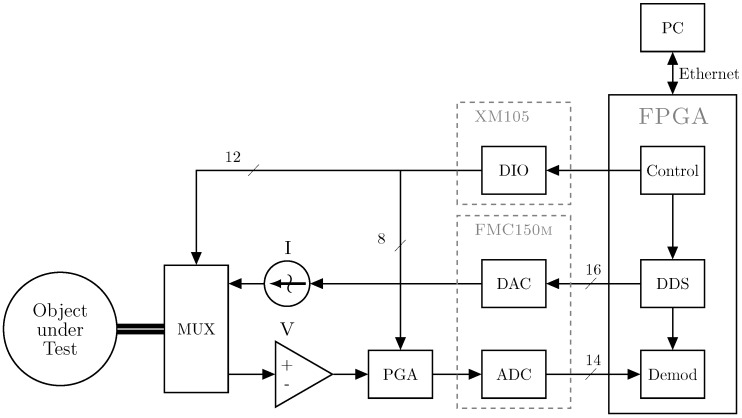
Simplified block diagram of the mfEIT (multi-frequency EIT) system.

Figure 2 gives a simplified block diagram of the system. The FPGA is responsible for the signal form generation of the current to be injected (Section 2.2) as well as for receiving and processing the measured voltages (Section 2.4). The adjacent method is used and performed by controlling multiplexers (MUX), which switch the electrodes for both current injection and voltage measurements (Section 2.3). To adjust the amplitude of the measured voltages, a programmable-gain amplifier (PGA) is controlled by the FPGA. For each measurement, the FPGA performs the demodulation of the received voltages (Section 2.4.1), and finally sends the demodulated data to the PC over the ethernet (Section 2.5).

### 2.1. Main Controller

The system is based on a *Virtex-6 FPGA DSP Kit with AD/DA* (Avnet^®^, Phoenix, AZ, USA), which includes a *Virtex-6 FPGA ML605 Evaluation Board* (Xilinx^®^, San Jose, CA, USA) and a *FMC150* analog digital board (4DSP^®^, Austin, TX, USA). A *XM105 Debug Card* (Xilinx^®^, San Jose, CA, USA) is used for digital input/output (DIO) connections. The FMC150 board was modified by changing the input and output transformers of the AC coupling to fit our frequency range requirements. After modification, the bandwidth of the analog input of the analog digital converter (ADC) is 0.03-125 MHz, and the bandwidth of the analog output of the digital analog converter (DAC) is 0.06-300 MHz. The FMC150 board includes a dual channel 14-bit ADC (ADS62P49/ADS4249, Texas Instruments, Dallas, TX, USA) and a dual channel 16-bit DAC (DAC3283, Texas Instruments, Dallas, TX, USA). The DAC is followed by a 82 MHz 5th order Chebyshev low-pass filter.

The designed hardware was programmed in very high speed integrated circuit hardware description language (VHDL) in the PlanAhead^TH^ 14.7 Tool. The CORE Generator was used to generate the basic cores, e.g., clocking wizard, Direct Digital Synthesizer (DDS), and the Cascaded Integrator Comb (CIC) filter, required for the design.

### 2.2. Signal Generation

For mfEIT, injecting multiple sine wave currents at different frequencies is necessary; this can be achieved by:

1. injecting two single currents of different frequencies at two separated electrode pairs simultaneously [19];
2. injecting single sine currents at different frequencies sequentially [20,21];
3. injecting a swept-frequency (chirp) waveform [22,23];
4. injecting a mixed waveform current, composed of multiple sine waves of different frequencies simultaneously [19,24,25].

Since our system only incorporates a single current source, the first method is not possible. Measuring by injecting currents at different frequencies sequentially (method 2) may not be a problem in a static dummy. However, when performing measurements at the torso, the lung inflates and deflates, and its impedance changes accordingly. If both measurements are not perfectly matched with the breath, additional errors may be introduced to the reconstructed images. Injecting a sweep of frequencies (method 3) seems promising, gaining the complete spectrum of frequencies in only one measurement. However, since this signal is non-stationary, the precise extraction of the amplitude and phase from the data is difficult [23]. Moreover, averaging the measurement over multiple periods for better signal-to-noise ratio (SNR) is not possible. For these reasons, injecting a mixed waveform current (method 4) was chosen and implemented in this work.

#### Multi-Sine Waveform Generator

The *Direct Digital Synthesizer (DDS) Compiler v5.0* [26] was used to create the look-up tables (LUT) of sine and cosine waves. Configured in a streaming phase incremental programability, this enables the use of only one core using multiple instances of it for different sine frequencies. Although the theory of DDS is well-known [27], we provide a supplement with some information for convenience to the reader.

In the presented EIT system, a system clock of 245.76 MHz and a phase accumulator of 16 bit were chosen, yielding a frequency resolution of 3750 Hz. If another resolution is required, the number of bits of the phase accumulator can be adapted accordingly.

Thus, in order to generate a sine wave of 120 kHz, an phase increment value of 32 has to be given.

The multi-frequency wave is then created by simply adding the output sine waves of each DDS instance. However, attention must be paid when summing up both sine waves, in order to avoid overflow. The multi-sine wave created for the current injection $I_{\mathrm{inj}}$ is represented by:

(1) $$\begin{array}{c} I_{\mathrm{inj}}=\sum_{i=1}^{n}A_{{\mathrm{ref}}_{i}}\cdot \sin\left(\omega_{i}t\right),A_{{\mathrm{ref}}_{i}}=A_{\mathrm{ref}} \end{array}$$

where *n* represents the number of frequency components, and *t* the time. The amplitude $A_{{\mathrm{ref}}_{i}}$ of each frequency component $w_{i}$ was set to be the same and equal to $A_{\mathrm{ref}}$.

**Figure 3:**
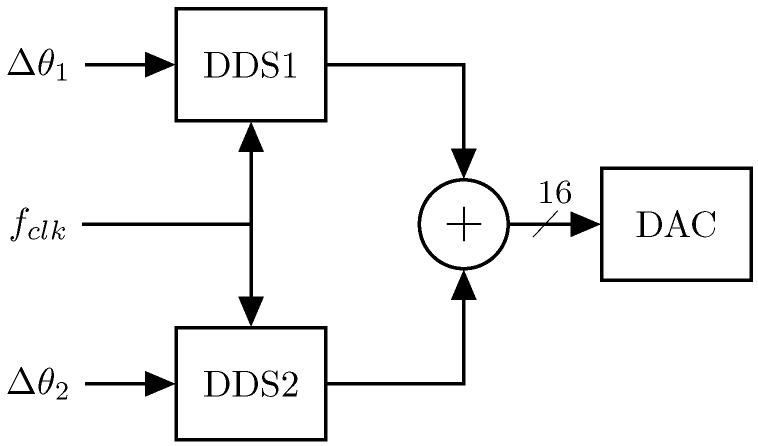
Multi-sine waveform generation for two frequencies.

The 2’s complement of the multi-sine waveform is finally transmitted to the 16-Bit DAC. The initialization and configuration of the DAC internal registers, e.g., gain, is done by SPI. Figure 3 shows the block diagram of the multi-sine waveform generator for n=2.

### 2.3. Electrode Multiplexing

**Figure 4:**
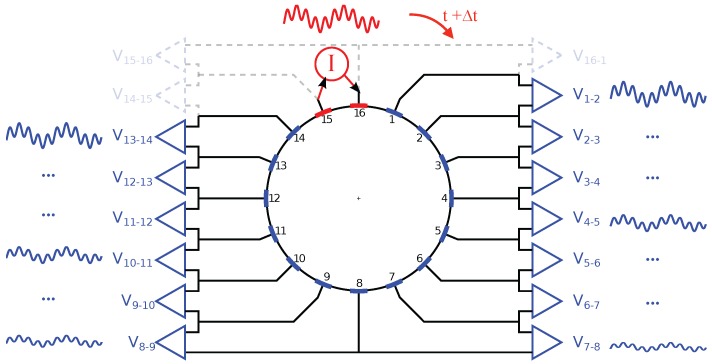
Adjacent method with injection of a composite waveform with two frequencies. Measurements at each electrode pair are performed sequentially. Measurements at electrode pairs containing the injection electrodes are not performed.

In this work, the adjacent measurement method [28], depicted in Figure 4, was implemented. A composite waveform is used for current injection at a pair of two adjacent electrodes, and the voltage measurements are performed between the other adjacent electrode pairs sequentially. After the complete measurement of this injection cycle, the injection pair is rotated by one electrode to the next adjacent pair and the measurement process is repeated. In a 16-electrode EIT system, when using the adjacent method and not measuring at the injecting electrodes, 16×13=208 voltage values are obtained for each complete measurement cycle.

Considering the number of current injections $N_{I_{i}}$, the number of voltage measurements $N_{V_{m}}$, and the number of oscillations of one (multi-)sinus burst $N_{\mathrm{osc}}$, the time of one measurement cycle $t_{m}$, when measuring at a frequency *f* is given by:

(2) $$\begin{array}{c} t_{m}\left[\mathrm{ms}\right]=N_{I_{i}}\cdot N_{V_{m}}\cdot N_{\mathrm{osc}}\cdot \frac{1}{f[\mathrm{kHz}]} \end{array}$$

**Figure 5:**
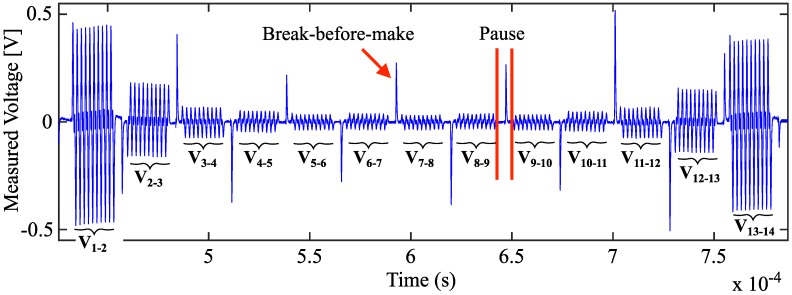
Measurement of one current injection cycle with injection in electrode pair 15–16 (as in Figure 4) performed at 480 + 960 kHz. Measurements of the remaining electrode pairs are marked with V${}_{1-2}$ to V${}_{13-14}$. The break-before-make interval provoked by the multiplexer is inside the implemented pause time.

Since our system incorporates only one current injection channel and one measurement channel, these signals must be multiplexed sequentially through all electrode pairs. We use four multiplexers, two for current injection and two for voltage measurements and, from these, two are connected only to the even and the other two only to the odd electrodes. To avoid the undesired break-before-make interval of the multiplexers—provoked by the switch breaking (opening) the first contacts before closing the new ones—affects the injected and measured signals, pauses between measurements were inserted (Figure 5). The time for the pauses $t_{p}$ of one measurement cycle is described by:

(3) $$\begin{array}{c} t_{p}\left[\mathrm{ms}\right]=(\left(N_{V_{m}}-1\right)\cdot N_{I_{i}}\cdot N_{P_{V}}+\left(I_{i}-1\right)\cdot N_{P_{I}}+1\cdot N_{P_{\mathrm{cycle}}}\cdot \frac{1}{f[\mathrm{kHz}]} \end{array}$$

where $N_{P_{V}}$ is the number of theoretical oscillations in a pause when switching the measuring electrode pair, $N_{P_{I}}$ is the number of theoretical oscillations in a pause when switching the injection electrode pair and $N_{P_{\mathrm{cycle}}}$ is the number of theoretical oscillations of the pause at the end of a complete cycle. The total time of a measurement cycle $t_{\mathrm{cycle}}$ is then given by the sum of these two times $t_{m}+t_{p}$.

When measuring in a multi-frequency mode, the measurement time is defined according to the lowest frequency component.

Our system is programmed to measure from 60 kHz up to 960 kHz. For a measurement with *f* = 90 kHz and $N_{\mathrm{osc}}$ = 10, giving a pause between measurements to suppress the multiplexing noise, we get a $t_{\mathrm{cycle}}$ = 30.4 ms, that corresponds to 32.8 fps for each demodulation component. When measuring at higher frequencies, the frame rate increases. If a higher frame rate is not necessary, the number of oscillations can be increased to improve the SNR.

### 2.4. Data Acquisition and Processing

The measured data from the 14-bit ADC is buffered and sign-extended to 16-bit format. The amplitude of the input signal is adjusted by using a PGA to avoid overflow. The gain of the PGA can be controlled manually, setting its gain to a fixed value, or it can be controlled automatically by the FPGA. The received data are demodulated using the phase-sensitive (I/Q) detector.

#### 2.4.1. Phase-Sensitive Demodulation

Phase-sensitive demodulation (PSD) is a simple and reliable demodulation method to calculate the in-phase (I) and quadrature (Q) components of the measured voltages. However, it needs two demodulation blocks for each frequency to be demodulated simultaneously, which can become costly in terms of FPGA resources if the number of frequencies acquired simultaneously is too high. A workaround is to perform the demodulation multiple times for the same data set [17].

Considering a number of frequencies *n*, the measured voltages ($V_{\mathrm{meas}}$) modulated by the body are represented by:

(4) $$\begin{array}{c} V_{\mathrm{meas}}=\sum_{i=1}^{n}A_{i}\cdot \sin\left(\omega_{i}t+\varphi_{i}\right) \end{array}$$

where $A_{i}$ is the modulated amplitude and $\varphi_{i}$ is the phase induced by the tissues impedances, for each frequency $w_{i}$.

The demodulation of the I and Q components for each frequency is performed separately by multiplying the measured signal $V_{\mathrm{meas}}$ with the original sine and cosine of each frequency, respectively. For that, a dedicated multiplier of a XtremeDSP Slice is used. Although the theory of PSD is well-known [29], we provide a supplement with the intermediate steps of the demodulation for convenience to the reader. The following equations show the generalized $I_{i}$ and $Q_{i}$ component of a frequency $w_{i}$.

(5) $$\begin{array}{ccc} V_{i,I} & = & \frac{A_{i}A_{\mathrm{ref}}}{2}\cdot \cos\left(\varphi_{i}\right)=I_{i} \end{array}$$

(6) $$\begin{array}{ccc} V_{i,Q} & = & \frac{A_{i}A_{\mathrm{ref}}}{2}\cdot \sin\left(\varphi_{i}\right)=Q_{i} \end{array}$$

**Figure 6:**
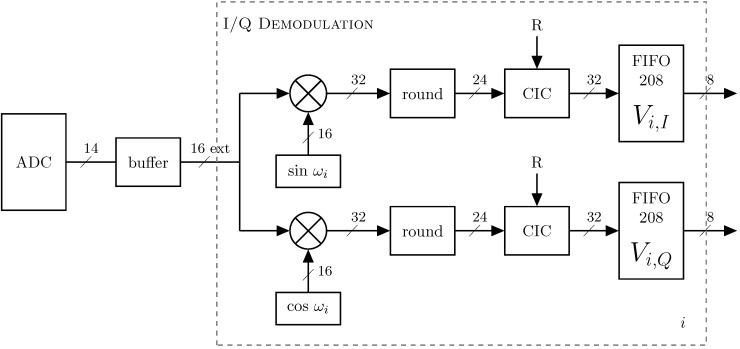
Simplified block diagram of the demodulation process of the multi-frequency EIT of the 16-bit signal extended measured voltages. The block is repeated for each frequency $\omega_{i}$, and the sample rate change is obtained by decimation by a factor R.

Figure 6 presents the compact block diagram of the demodulation implemented in the FPGA for both *I* and *Q* components, of the 16-bit signal extended (16 ext) measured voltages. This block is repeated for each frequency $\omega_{i}$.

**Figure 7:**
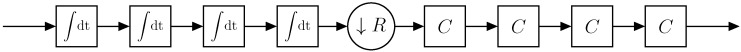
CIC four-stage decimator: ∫—integrator stages, ↓R—decimation by a factor *R*, *C*—comb stages.

A Cascaded Integrator-Comb (CIC) filter was used for low-pass filtering. CIC filters are similar to moving average filters in which, in each step, the filter is implemented recursively, adding the next sample and subtracting the last. They are a special class of digital linear-phase finite impulse response (FIR) filters used for decimation and interpolation [30]. It is advisable to use CIC filters as an alternative to other FIR filters, when high sample rate changes are desired. Figure 7 shows a 4-stage CIC decimator. It has four cascaded integrator stages, followed by a decimator of factor R (sample rate change) that, in turn, is followed by four comb stages. The CIC filters only use additions and subtractions, and do not employ multipliers [30]; this is why they are a good option for FPGA implementations.

As the CIC core of the FPGA only allows signals of 24-bit maximum, the precision of the multiplication result (32-bit) had to be reduced (rounded). The CIC was configured as one channel decimation filter with four stages *N* and a differential delay *M* = 1. The sample rate change *R* was set to be programmable depending on the frequency used, and has an absolute maximum of 8192 samples. The window has to contain an integer number of complete periods of the frequency $\omega_{i}$. The output width was fixed to 32-bit using truncation quantization.

Sequentially, the resultant I and Q components are saved in separated FIFOs, each containing the 208 values of 32-bit corresponding to one complete cycle, i.e., one EIT frame.

**Figure 8:**
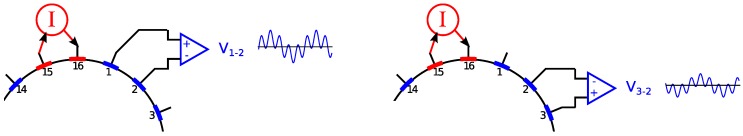
Inversion of the measured signals due to the differential amplifier, with current injection in electrode pair 15-16. On left, measurement between electrodes 1 and 2 (“V${}_{1-2}$”); on right, measurement between electrodes 2 and 3 (“V${}_{3-2}$”).

As mentioned in Section 2.3, our system makes uses of multiplexers and two of them are connected only to the even electrodes and the other two only to the odd electrodes. Furthermore, the differential amplifier connected to the output of the multiplexers has its positive pole connected to the multiplexer of the odds electrodes and the negative pole connected to the one of even electrodes. Hence, when measuring between electrode 1 and 2 (“V${}_{1-2}$”), and after between 2 and 3 (“V${}_{3-2}$”), the signal of the second measurement will be inverted relative to the first one as depicted in Figure 8, and so forth. Thus, due to the difference amplifier, in every second voltage measurement the signal is inverted. Because of this system configuration, we are limited in the combinations of measurements we can perform, and thus this influence has to be corrected.

Analogue to the current injection pair, in which the even and odd electrodes are connected to $180^{\circ }$ (*π* radians) phase shifted signals, working one as source and the other as sink, and therefore the injected signal is inverted for every second electrode pair. When demodulating an inverted measured signal, using a non-inverted reference signal, the I and Q components will be inverted:

(7) $$\begin{array}{ccc} V_{i,I} & = & \frac{A_{i}A_{\mathrm{ref}}}{2}\cdot \left(-\cos\left(\varphi_{i}\right)\right)=I_{i} \end{array}$$

(8) $$\begin{array}{ccc} V_{i,Q} & = & \frac{A_{i}A_{\mathrm{ref}}}{2}\cdot \left(-\sin\left(\varphi_{i}\right)\right)=Q_{i} \end{array}$$

**Figure 9:**
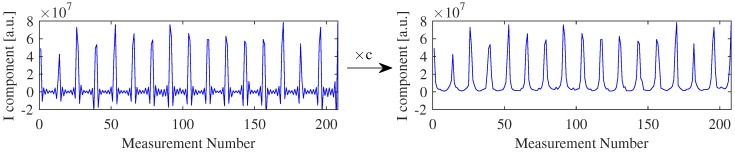
Correction of the signal inversion during measurements. Left, a sample of the I component of the raw data acquired, with inverted signals. Right, the corrected signal (typical “U-Curves”) obtained by multiplying the raw data by the correction array *c*.

The correction of this signal inversion was performed in the processing after the demodulation by the software at the PC. For that, the 208 values of the I and Q components were corrected by multiplying these signals with an array containing 208, −1 or 1 values, since $\sin\left(\omega_{1}t+\pi \right)=-\sin\left(\omega_{1}t\right)$ and $\cos\left(\omega_{1}t+\pi \right)=-\cos\left(\omega_{1}t\right)$. Only the values multiplied by −1 are inverted. The correction array *c* is a combination of both, the inversion provoked by the voltage measurement and the inversion provoked by the current injection. Figure 9 is an example of the correction of the signal inversion.

To consider the interaction between the conductivity (in-phase) and permittivity (quadrature) of the measured signals (whose inversion was previously corrected), the amplitude (*A*) and the phase (*φ*) were calculated in Matlab and were obtained from the following equations:

(9) $$\begin{array}{ccc} |V_{i}| & = & \sqrt{Q_{i}^{2}+I_{i}^{2}}=\frac{A_{i}\cdot A_{\mathrm{ref}}}{2}\Rightarrow A_{i}=\frac{|V_{i}|\cdot 2}{A_{\mathrm{ref}}} \end{array}$$

(10) $$\begin{array}{ccc} \varphi_{i}\left[\mathrm{rad}\right] & = & \arctan\frac{Q_{i}}{I_{i}} \end{array}$$

In Matlab, the phase is calculated using the function *atan2* or *angle*, which returns the four-quadrant inverse tangent, in combination with the function *unwrap* to correct the radian phase angle jumps.

As frequency-difference images of amplitude as well as phase are intended, further attention is required when treating the phase component. The resultant phase $\varphi_{i}\left[\mathrm{rad}\right]$ is frequency-dependent. Thus, it must first be converted to time shift $\Delta t_{i}$ in seconds, in order to be comparable between frequencies:

(11) $$\begin{array}{ccc} \Delta t_{i}\left[s\right] & = & \frac{\varphi_{i}\left[\mathrm{rad}\right]}{w_{i}},w_{i}=2\pi f_{i} \end{array}$$

To suppress the amplitude and phase dependencies at different frequencies of the measurement device itself, the data are normalized. For the amplitude at each frequency ($A_{i}$), the amplitude is normalized by its maximum of the 208 values:

(12) $$\begin{array}{c} A_{i,\mathrm{norm}}=\frac{A_{i}}{A_{i,\max}} \end{array}$$

so that all the values are between a value near 0 (but not 0) and 1.

The phase, in this case as time shift, is normalized by subtracting the mean value of the time shift ($\bar{\Delta t_{i}}$) of a frame (208 measurement values), so that the relative time shifts in seconds relative to its mean stay the same. Only the offset time shift, generated mostly by the measurement system itself, is canceled:

(13) $$\begin{array}{c} \Delta t_{i,\mathrm{norm}}\left[s\right]=\Delta t_{i}-\bar{\Delta t_{i}} \end{array}$$

Thus, the time shifts for both frequencies are centered at 0.

Finally, the frequency-difference data of the amplitude ($d_{A}$) and time shift ($d_{\Delta t}$), necessary for the reconstruction, is given by:

(14) $$\begin{array}{ccc} d_{A} & = & A_{f_{2},\mathrm{norm}}-A_{f_{1},\mathrm{norm}} \end{array}$$

(15) $$\begin{array}{ccc} d_{\Delta t} & = & \Delta t_{f_{2},\mathrm{norm}}-\Delta t_{f_{1},\mathrm{norm}} \end{array}$$

where $f_{1}$ is the lowest frequency, and the $f_{2}$ the highest one.

#### 2.4.2. Increasing the Resolution of the ADC-Channel

Normal averaging performed by summing up *n* samples and dividing the result by *n* acts as a low-pass filter attenuating the noise and flattening out peaks of the input signal. However, it does not increase the resolution of the conversion. An increase of resolution can be achieved by combining oversampling with the averaging method decimation (or interpolation) [31].

Oversampling is a method to increase the number of effective bits of an ADC and, thus, its resolution, without the need to acquire a more expensive ADC of higher resolution [32]. Oversampling is achieved by sampling a signal with much higher sampling frequency than the Nyquist rate. Together with the increase of resolution, oversampling also reduces the noise. Generally, the effective SNR of an ADC is given by:

(16) $$\begin{array}{c} \begin{array}{c} {\mathrm{SNR}}_{\mathrm{dB}}=\left(6.02\cdot \mathrm{ENBO}\right)+1.76 \end{array} \end{array}$$

where ENBO is the effective number of bits.

For each doubling of the oversampling rate, 3 dB or half bit of resolution is gained [32]. The SNR of an ideal 14-bit ADC is 86.04 dB. In our system, using a sampling frequency of 245.76 MHz, if we consider a maximum input signal of 1 MHz, and thus a Nyquist frequency of 2 MHz, an oversampling factor of 122.88 is achieved, which is equivalent to 3.47 extra bits of resolution. Using this oversampling rate, an ideal 14-bit ADC would provide a 17.47-bit resolution or a SNR of 106.9 dB. Hence, 106.9 dB is the theoretical upper limit for SNR in our system, and might not be reached in actual practice.

### 2.5. Data Transfer

The FIFO, in which the data of the demodulation is saved, is configured as native interface type using independent clocks for write and read operations. Herewith, different aspect ratios for the data ordering are possible. For the write data port, a clock of 245.76 MHz (the same used for the signal generation) was used, and for the read data port, a 125 MHz clock (the same used in the ethernet connection) was employed. We configured a 4:1 aspect ratio, so that we have 32-bit input width and a reordered output of a width of 8-bit, ready for the ethernet transfer.

The ethernet connection was implemented using the IP-Core *Virtex-6 Embedded Tri-Mode Ethernet MAC Wrapper.* This core allows different configurations of the PHY Interface. A connection using the SGMII with 10/100/1000 Mbps and PHY auto-negotiation enabled was configured. The user datagram protocol (UDP), including source and destination internet protocol (IP) addresses and ports definitions, was implemented in VHDL.

The ethernet connection was used to transfer the demodulated data from the FPGA to the PC. Each demodulation component of each frequency, together with corresponding headers, is sent sequentially in separated ethernet packets in big-endian format. Additional information, such as the used gain of the PGA for the input signals in the ADC, is also sent to the PC.

#### 2.5.1. Data Rate

The total time of a measurement cycle, i.e., the time necessary to generate data for one EIT frame, is given by the sum of Equations (2) and (3). Considering $N_{P_{V}}=3$, $N_{P_{I}}=5$, $N_{P_{\mathrm{cycle}}}=10$ and $N_{\mathrm{osc}}=10$ oscilations, and considering the lowest frequency of the used frequency pair *f* = 90 kHz, approx. 132 ethernet packets per second, each of them with 1024 byte of data (plus UDP header) are sent to the PC, which corresponds to 1,125,696 bits/s = 1.1 Mbit/s. Since these 132 ethernet packets contain four demodulation components (using *n* = 2 frequencies), this corresponds to approx. 33 EIT frames/s. When measuring at higher frequencies, these data rates also increase. For a frequency of 360 kHz, 525 ethernet packets/s, corresponding to 131 EIT frames/s are generated, and 4.5 Mbit/s data are transferred. However, software at the PC cannot usually handle such high data quantity and also reconstruct the figures in real-time. Nevertheless, they can be saved at this data rate for offline analysis and it is recommended to use downsampling for online visualization.

#### 2.5.2. Multi-Frequency EIT Software

**Figure 10:**
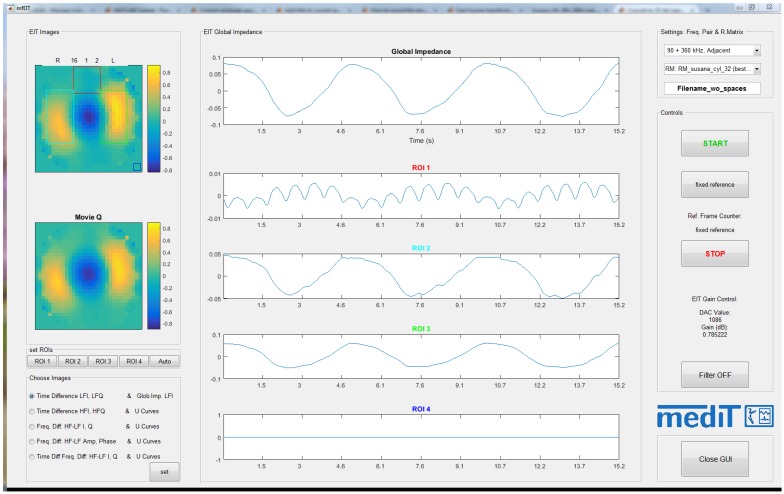
Screen capture of the multi-frequency EIT software.

The ethernet connection is also used for the PC to communicate with the FPGA through the developed mfEIT software. From the software, control settings (e.g., to set the measurement frequencies, or to start/stop the measurements) are possible. Figure 10 shows a screen capture of the graphical user interface of the developed software. On the left lower corner, different modes of reconstruction and display can be chosen.

With this software, we can display the reconstructed time-difference images of both I and Q components for each frequency in real-time, see the global impedance curve (averaged) as well as the pre-defined regions-of-interest (ROIs), or redefine the ROIs to visualize the impedance curves of a desired region. Additionally, frequency-difference images of amplitude and time shift are also available. The so-called ’U-curves’ of each component, and computed amplitude and time shift curves, can also be displayed. Furthermore, it is possible to change between some of the reconstruction matrices used.

## 3. Measurement Setup

To evaluate the system, measurements were carried out by injecting a current at two different frequencies simultaneously in a composite waveform, and measuring the resulting induced voltages. Time-difference and frequency-difference images were performed using a resistor test unit with capacitances as inhomogeneity, and also in a self-experiment.

### 3.1. Resistor/Capacitor Test Unit

**Figure 11:**
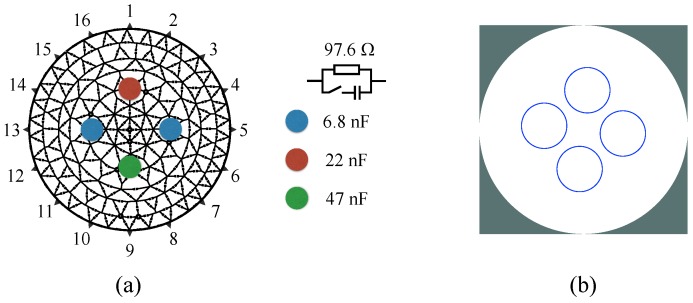
Resistive/capacitive test unit: (**a**) with the (marked) positions of the inhomogeneities and respective values of the used capacitors; (**b**) approximated ground truth for reconstructed images.

Figure 11a shows the configuration of the resistive test unit, with switchable capacitors placed in parallel to a resistor marked with colored circles. Although not visible in the image, in each electrode an extra cole cell (R || RC) is present to mimic the electrode impedance. Figure 11b shows the approximated ground truth of the reconstructed images for this test unit.

### 3.2. Human Experiments/Self-Experiment

**Figure 12:**
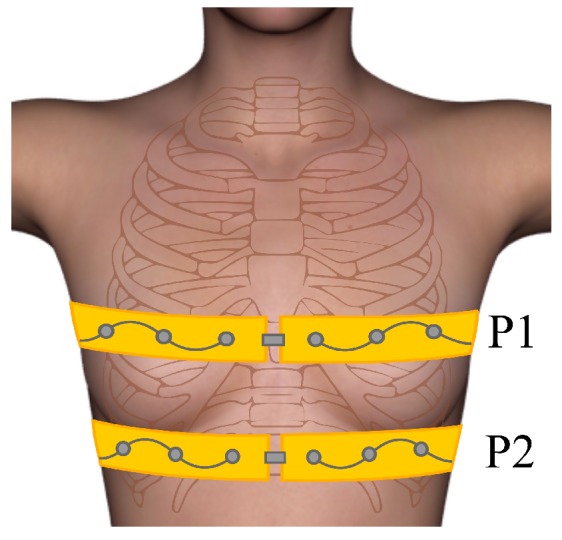
Approximation of the P1 and P2 positions of the electrode belt fixed on the volunteer’s thorax.

The measurements were performed on one healthy female volunteer, 28-years-old, with a body mass index (BMI) of 19.1 kg/m^2^. A 16-electrode belt (Dräger, Lübeck, Germany) was placed on the subject’s thorax at two different positions (approximately P1 and P2) as shown in Figure 12. The new mfEIT prototype was used to perform the measurements, using different sets of two frequencies. The amplitude of the injected current was set to be within electrical safety standards (IEC 60601-1-1 2005).

## 4. Results

The results of the first measurements performed with the mfEIT prototype are presented. The reconstruction of both the time-difference and frequency-difference images are performed in Matlab using the GREIT algorithm [33] included in the EIDORS toolbox. In order to better evaluate the system, simulations of the resistive/capacitive test unit were performed in Matlab/Simulink and are also presented.

### 4.1. Resistive/Capacitive Test Unit

**Figure 13:**
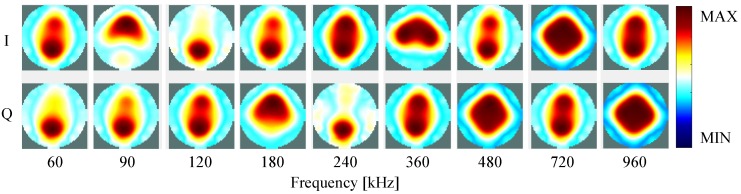
Time-difference images of the test unit using the inhomogeneities at the positions shown in Figure 11, for both real (I) and imaginary (Q) components of measurements at different frequencies. Note that the figures are all autoscaled individually for better visualization, and hence the color-scale only shows MAX and MIN, which is correspondent to the maximum and minimum value of each figure.

Figure 13 shows the reconstructed real and imaginary components (I and Q, respectively) of time-difference images of the measurements on the resistive/capacitive test unit performed at different frequencies. The test unit in the homogeneous state, i.e., with all the four capacitors switched off, was used as reference. In the inhomogeneous state, all the four capacitances were switched on in parallel with the respective resistor.

Significant differences on the reconstructed images were detected between the different inhomogeneities by measuring at different frequencies using the new mfEIT prototype. If in Figure 13 from 60 kHz–240 kHz only the top and bottom part, where the inhomogeneities with the 22 nF and 47 nF capacitors are placed, are visible (partly in the component I and partly in Q), at frequencies from 360 kHz both left and right inhomogeneities (6.8 nF) start to be also apparent. Both real and imaginary components seem to provide important information about the inhomogeneities. However, caution is required when looking at these components separately since they interact with each other. Hence, the amplitude and time shift is also computed and analyzed.

**Figure 14:**
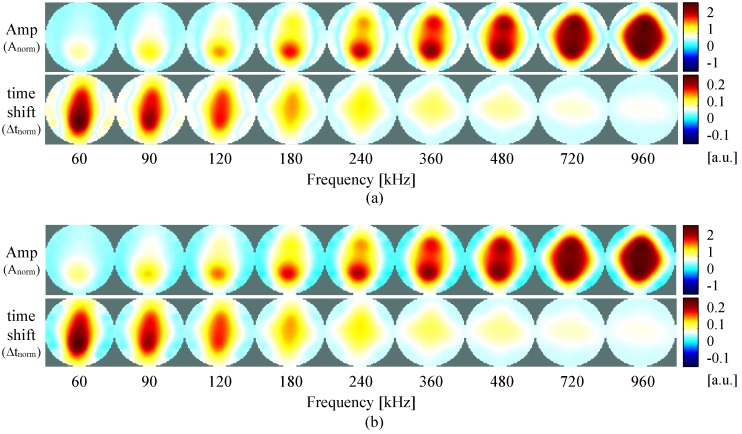
Time-difference images of the test unit measurements (**a**) and simulation (**b**) using the inhomogeneities at the positions shown in Figure 11, for both amplitude and time shift of measurements at different frequencies.

In Figure 14, results of time-difference imaging are present. In this figure, the measurements at each frequency are presented in the same color-scale. As so, depending of the frequencies, some of the perturbations are not visible since they are much weaker than on other frequencies. In order to better evaluate the system, and understand the results of the measurements (Figure 14a), simulations of the same test unit are depicted in Figure 14b.

Analyzing both results of measurements and simulations, no significant differences are found. This proves that our system performs well and is accurate in the tested frequency range. At 60 kHz, in the amplitude figures only the bottom inhomogeneity is lightly visible, while in the time shift figures the inhomogeneity extends from top to bottom. When increasing the frequency to 360 kHz, both top and bottom inhomogeneities are visible in the amplitude, while in the time shift the perturbation extends to the four inhomogeneities (top, bottom, left and right) but is only lightly visible. If one increases the frequency to 960 kHz, the perturbation visible in the amplitude extends to the four places where the inhomogeneities are placed, while in the time shift it is now only lightly visible on left and right. For better visualization and interpretation of the behavior at each frequency, the amplitude and time shift for the measurements, reconstructed and color-scaled individually, is given in the supplementary file (Figure S1).

**Figure 15:**
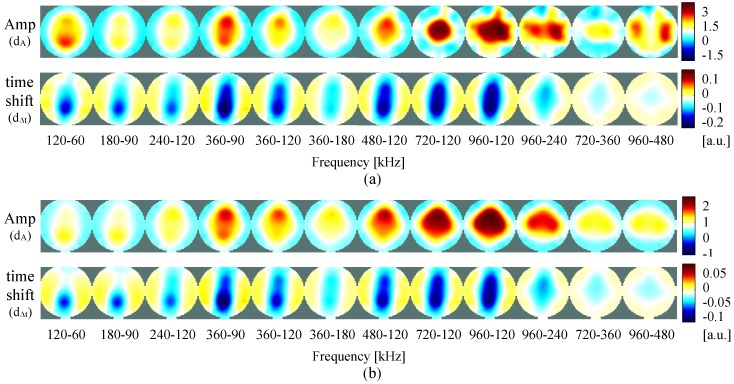
Frequency-difference images of the test unit measurements (**a**) and simulation (**b**) using the inhomogeneities at the positions shown in Figure 11, performed at different frequency pairs.

Figure 15 shows the frequency-difference images of the computed amplitude and time shift of the measurements on the test unit (a) and of the simulations (b), performed at different combinations of frequency pairs. Here, no homogeneous reference is used. The reconstructed images are computed directly by calculating the difference between the measurements at the different frequencies, in which the measurement at lower frequency is set as reference (Equations (14) and (15)).

**Figure 16:**
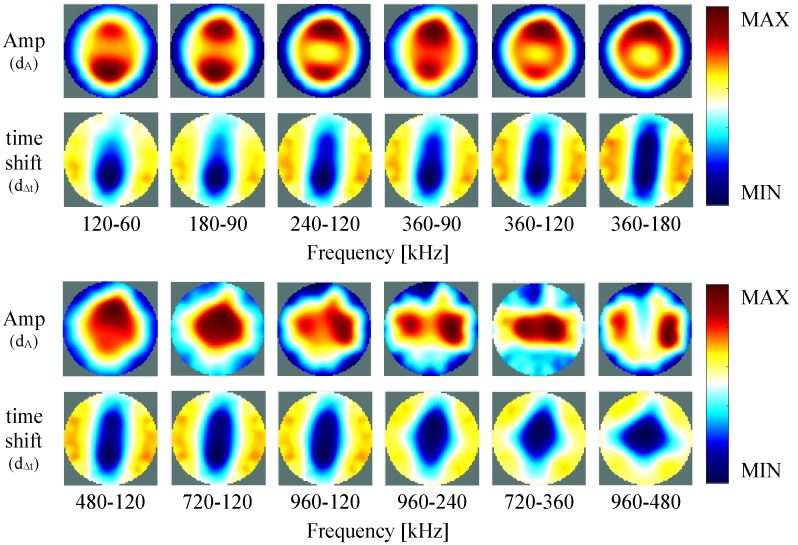
Frequency-difference images of the test unit using the inhomogeneities at the positions shown in Figure 11, performed at different frequency pairs. Note that all the figures are reconstructed separately and are autoscaled individually for better visualization. The MAX and MIN of the color-scale is correspondent to the maximum and minimum value of each figure.

In Figure 15, the figures are depicted in the same color-scale. Here, small differences between measurements and simulation results are visible. This difference might be due to the normalization process, that might be causing loss of information in the measurement results. To improve visualization, in Figure 16 the results for the measurements with each figure color-scaled individually are also presented. Here, the color-scale only shows MAX and MIN, since is correspondent to the maximum and minimum value of each figure individually. As expected, depending on the frequency pair used for the measurement, different inhomogeneities can be detected. Generally, in frequency-difference imaging, a low frequency at the Hz range is used as reference. However, for our measurements we did not always use the same frequency as reference, but also tried other combinations. As can be seen, some inhomogeneities are detected more clearly when using higher frequencies as reference. In this case, the left and right inhomogeneities are stronger visible when the lowest frequency used was higher than 240 kHz.

When performing the difference between two measurements at the lowest frequencies 120 kHz–60 kHz, perturbations are most visible on the bottom part for the amplitude and the time shift. When increasing both frequencies to 360 kHz–120 kHz, the inhomogeneities are most visible on the top for the amplitude, and similar on top and bottom for the time shift. Increasing further the higher frequency and measuring between 720 kHz–120 kHz, the perturbation appears at the center, so that all the four inhomogeneities seems to contribute equally for the perturbation in amplitude, however, in the time shift it is still visible only on top and bottom. If one increases both frequencies further, by measuring at 960 kHz–480 kHz, only the left and right inhomogeneities are visible at the amplitude signal, and by the time shift, all four inhomogeneities seems to contribute equally to the perturbation. Although these measurements are only obtained from a resistive/capacitive test unit and therefore do only partly mimic the human body, it seems important to try other frequency combinations (other than those with a reference < 100 kHz only).

### 4.2. Self-Experiment

**Figure 17:**
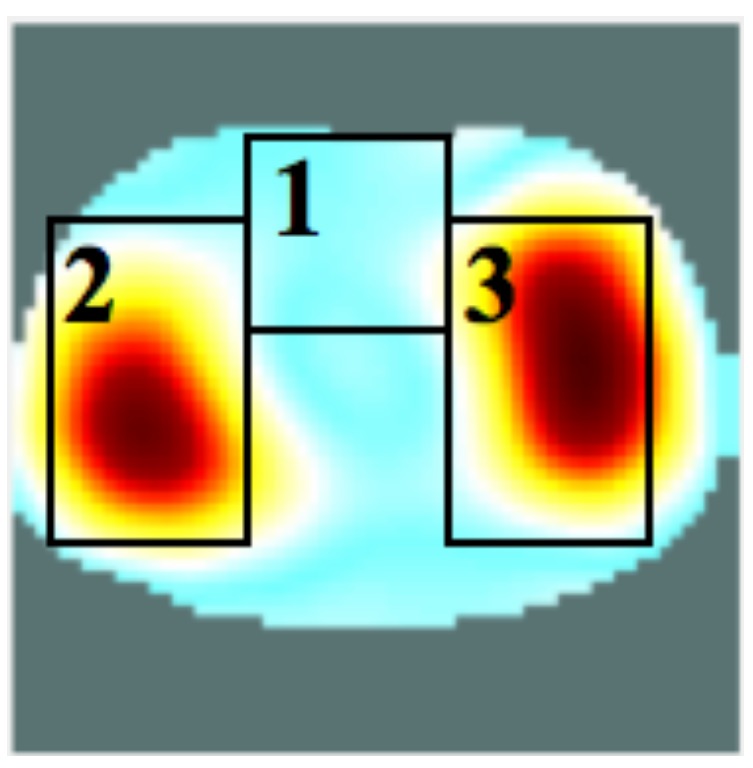
Default regions-of-interest. ROI 1: heart region, ROI 2: right lung region, ROI 3: left lung region.

**Figure 18:**
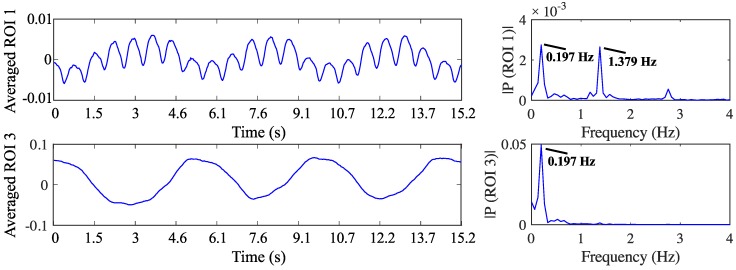
Bio-signals of the volunteer: (**Left, upper**) average of the pixel values of ROI 1, corresponding to the heart region; (**Right, upper**) corresponding amplitude spectrum of the signals of ROI 1; (**Left, lower**) average of the pixel values of ROI 3 corresponding to the left lung region; (**Right, lower**) corresponding amplitude spectrum of the signals of ROI 3.

**Figure 19:**
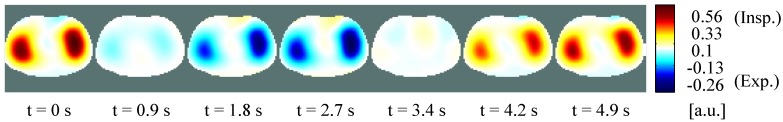
Time-difference images of one respiration cycle, measured at 90 kHz.

Figure 17 shows a classical functional EIT image including the ROIs corresponding to the heart region (ROI 1), and the right (ROI 2) and left (ROI 3) lung regions. Figure 18 presents the bio-signals corresponding to the ROI 1 (heart) and ROI 3 (left lung). The signals were obtained from a measurement performed at 90 kHz and 360 kHz, and the presented curves correspond to the real-part of the 90 kHz measurement. The heart rate was confirmed by measurement with a pulse oximeter on the right index finger of the volunteer (during this measurement this was between 81–83 beats/min). On the amplitude spectrum presented in Figure 18 (right), two peaks at the frequencies 0.197 Hz and at 1.379 Hz were found, which correspond to the respiratory rate (11.82 breaths/min) and heart rate (82.74 beats/min), respectively. In Figure 19 the variation in lung impedance during one respiration cycle is shown.

**Figure 20:**
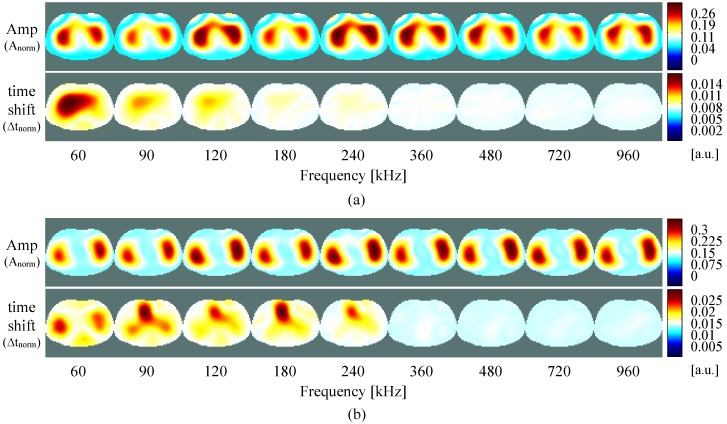
Functional images for both amplitude and time shift of the measured signals at the P1 (**a**) and P2 (**b**) positions of the electrode belt at diverse frequencies.

Figure 20 shows the functional images of the amplitude and time shift for measurements at each frequency, for the belt positions P1 (a) and P2 (b). These time-difference images provide information about any physiological processes that cause relatively fast changes in impedance, e.g., spontaneous breathing or ventilation, and heart beat. As can be seen, the mfEIT prototype effectively detects these ventilation-induced changes in the amplitude of the impedance at all used frequencies, for both the belt positions P1 (a) and P2 (b). The difference of signal strength between frequencies might be partly caused by the normalization process and by the slightly different spontaneous respiration cycles during the different measurements. Relative to the time shift signal, on belt positions P2 (b), strong perturbations seem to appear in the heart region at lower frequencies. An exception is the 60 kHz, in which the frame rate is too low, and is not enough for proper determination of cardiac signal. At higher frequencies, the perturbations in the heart region are much weaker than at lower frequencies, and so that not visible.

In belt position P1 (a) this behavior is sightly different, and might be explained by the different positioning of the belt, combined with the used frequency, and therefore the path that the current flows is different, being more or less affected by the cardiac signal. Hence, more measurements in different persons, belt positions and better normalization processes are necessary, in order to confirm if this is repeatable, in which conditions and hence if it is a meaningful information. The time-difference images of the I and Q components are also provided in the supplementary file (Figure S2).

**Figure 21:**
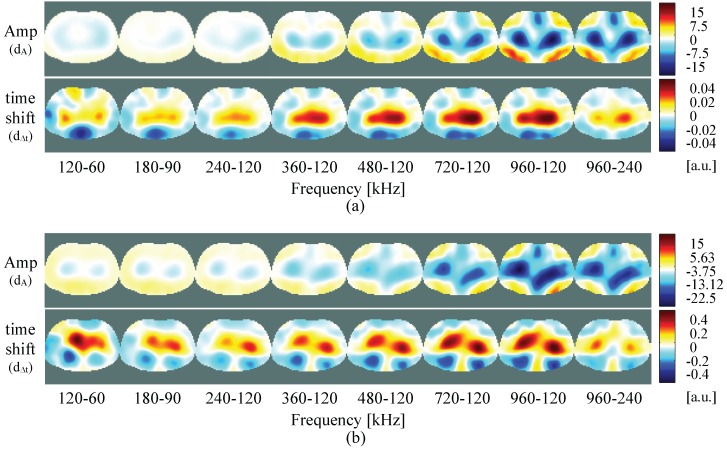
Frequency-difference images of the P1 (**a**) and P2 (**b**) positions of the electrode belt. Note that all the figures are in the color-scale.

The preliminary results of frequency-difference images of the measurements at different combinations of frequencies are presented in Figure 21, for the belt positions P1 (a) and P2 (b). For comparison, all figures are in the same color-scale. The frequency-different images color-scaled individually are present in the supplementary files (Figure S3), where the lowest frequencies can be better observed. In Figure 21 in the amplitude, regions at the lungs or muscle at the dorsal part seem to appear, depending on the frequency pair used for the measurement. Measurements at position P1 might be more accurate than at P2, since at position P2 the belt was located much lower, and some current might already be passing to the organs below the diaphragm. When looking to the time shift images of P1, perturbations at the lung positions also seem to be present. Furthermore, at the dorsal part a circular form perturbation appears, that might be provoked by the spinal cord. The time delay occurring when an electric current passes a cell membrane is expressed as phase angle. The phase angle is an indicator of the absence or presence of cell membranes with or without fluid [34]. As phase values are very small, the problem of getting accurate images is even bigger. We have normalized phase to time shifts, to allow comparisons. We believe that time shift images must be studied, since they might be a good indicator of the status of the cell membranes, and hence give some extra diagnostic information.

Nevertheless, because the reconstruction of the frequency-difference images is still preliminary, caution is required when interpreting these images. For the reconstruction of these images, simple frequency-difference was used. Furthermore, also the normalization procedure used for the data for each frequency might be leading to loss of information, and will be further investigated. Additionally, as expected, different positioning of the electrode belt produced different results. Thus, when interpreting the reconstructed images, the position of the belt must be taken into account.

## 5. Discussion and Conclusions

We have developed a novel 16-electrode multi-frequency EIT prototype, configured to measure impedance distribution in a cross-section of the body at different frequencies simultaneously by means of a composite waveform. The system employs a field-programmable gate array (FPGA) for low level control, which is programmed in a flexible way, so that some basic configurations can easily be changed via the graphical user interface, without the need for reprogramming the FPGA. The phase-sensitive demodulation of the measured data is performed directly in the FPGA and is subsequently sent to a PC via the ethernet, where the data is further processed and computed, and time-difference and frequency-difference images are reconstructed. Diverse frequency pairs for the measurements can be selected at the developed graphical user interface, and diverse signals can be plotted for detailed interpretation using the selected ROIs.

Some pilot measurements were carried out on a resistive/capacitive test unit and on a healthy volunteer; the time-difference results show that the new mfEIT prototype measures well at all tested frequencies from 60 kHz–960 kHz in static and dynamic cases. However, further analysis and improvement of the reconstruction algorithms is necessary to correctly reconstruct and posteriorly interpret the frequency-difference images. Furthermore, also the normalization procedure needs to be optimized, to minimize the loss of information. It is important to keep in mind that frequency-difference images, not using any reference in time, will contain information not only on lung and heart tissue, but also on other tissues (e.g., muscles, bones, fat) and all body parts. Ventilation and perfusion are noise for frequency-difference images, and hence must be filtered out. As far as we know, no frequency-difference images of humans or animals have been presented before. Although multi-frequency measurements have been performed by other groups, frequency-difference images have so far been only available from static phantoms or simulations, and only time-difference images at different frequencies of volunteers or animal trials have been presented.

Furthermore, positioning of the belt is important for correct imaging, since a too low position might detect movement provoked by the diaphragm, or even reach the liver or stomach regions [35]. Additionally, frequency-difference images seem to be very sensitive to electronic and technical problems, such as faulty cables or electrodes, so that we plan to implement algorithms using weighted frequency-difference to eliminate the systematic measurement artifacts, and develop new algorithms for further improvement of frequency-difference images.

Our FPGA-based system allows variation of the system without changes (or only minor changes) to the analog hardware. The parallel processing of an FPGA makes the system extremely fast and programmable. The frame rate of our system (depending on the frequency used for the actual measurement) may reach, e.g., at 360 kHz, 131 EIT frames/s. The presented system combines multi-frequency measurements for frequency-difference imaging with high frame rate time-difference imaging in a cost-effective way. The system was primarily developed focused in regional lung ventilation imaging and frequency-difference imaging. However, we did not want to restrict the device to it, but rather develop a combined device able to measure at high frame rates, so that in the future, we would be able to perform perfusion monitoring using the same device. Further upgrades of the device are planned, such as implementation of different measurement patterns, a demodulation by means of Fast-Fourier transform for comparison with the phase-sensitive demodulation, and triggers for interoperability with other medical devices. 

## Supplementary Materials

The supplementary materials are available online at www.mdpi.com/1424-8220/16/8/1158/s1.
