# Supplementary material for: System Description and First Application of an FPGA-Based Simultaneous Multi-Frequency Electrical Impedance Tomography
Source: Sensors (Basel). 2016 Jul 25;16(8):1158. doi: 10.3390/s16081158 (PMC5017324; doi:10.3390/s16081158)
Supplement: Supplementary file 1 [file sensors-16-01158-s001.pdf]

Supplementary Materials: System Description and First Application of an FPGA-Based Simultaneous Multi-Frequency Electrical Impedance Tomography

Susana Aguiar Santos, Anne Robens, Anna Boehm, Steffen Leonhardt and Daniel Teichmann

1. Direct Digital Synthesis

Direct Digital Synthesis (DDS) was used to create the sine and cosine waveforms. The desired output frequency f_{out} is set by changing the value of the phase accumulator and can be calculated from

$$f_{\text{out}} = \frac{f_{\text{clk}} \cdot \Delta\theta}{2^{B_{\theta(n)}}} \Rightarrow f_{\text{out}} = \Delta\theta \cdot a \tag{1}$$

where f_{clk} is the system clock frequency, $\Delta\theta$ is the phase increment value and $B_{\theta(n)}$ is the number of bits of the phase accumulator. The constant a gives the frequency resolution. In the presented EIT system, f_{clk} = 245.76 MHz and $B_{\theta(n)}$ = 16 bit were chosen, yielding a frequency resolution of 3750 Hz. If another resolution is required, the number of bits of the phase accumulator can be adapted accordingly.

Thus, in order to generate a sine wave of 120 kHz, a $\Delta\theta$ = 32 has to be given

$$\Delta\theta = \frac{f_{\text{out}}}{a} = \frac{120000\ \text{Hz}}{3750\ \text{Hz}} = 32 \tag{2}$$

2. Phase-Sensitive (I/Q) Demodulation

Phase-sensitive demodulation (PSD) is a simple and reliable demodulation method to calculate the in-phase (I) and quadrature (Q) components of the measured voltages.

Considering a number of frequencies n, the measured voltages (V_{meas}) modulated by the body are represented by:

$$V_{\text{meas}} = \sum_{i=1}^{n} A_i \cdot \sin(\omega_i t + \varphi_i) \tag{3}$$

where A_i is the modulated amplitude and φ_i is the phase induced by the tissues impedances, for each frequency w_i.

The demodulation of the I and Q components for each frequency is performed separately by multiplying the measured signal V_{meas} with the original sine and cosine of each frequency, respectively. For that, a dedicated multiplier of a XtremeDSP Slice at the FPGA is used. Considering the number of frequencies n = 2, the I and Q components for the measured voltage at frequency ω_1 (V_1) are obtained by:

$$V_{1,I} = (A_1 \cdot \sin(\omega_1 t + \varphi_1) + A_2 \cdot \sin(\omega_2 t + \varphi_2)) \cdot (A_{\text{ref}} \cdot \sin(\omega_1 t)) \tag{4}$$

$$V_{1,Q} = (A_1 \cdot \sin(\omega_1 t + \varphi_1) + A_2 \cdot \sin(\omega_2 t + \varphi_2)) \cdot (A_{\text{ref}} \cdot \cos(\omega_1 t)) \tag{5}$$

which is equivalent to:

$$\begin{aligned} V_{1,I} =& \frac{1}{2}(A_1 A_{\text{ref}} \cdot \cos(\varphi_1) - A_1 A_{\text{ref}} \cdot \cos(2\omega_1 t + \varphi_1) + \\ &+ A_2 A_{\text{ref}} \cdot \cos(\omega_2 t + \varphi_2 - \omega_1 t) - A_2 A_{\text{ref}} \cdot \cos(\omega_2 t + \varphi_2 + \omega_1 t)) \end{aligned} \tag{6}$$

$$V_{1,Q} = \frac{1}{2}(A_1 A_{\text{ref}} \cdot \sin(\varphi_1) + A_1 A_{\text{ref}} \cdot \sin(2\omega_1 t + \varphi_1) + \\ + A_2 A_{\text{ref}} \cdot \sin(\omega_2 t + \varphi_2 - \omega_1 t) + A_2 A_{\text{ref}} \cdot \sin(\omega_2 t + \varphi_2 + \omega_1 t)) \tag{7}$$

When low-pass filtering or integrating these signals for a complete period of the respective frequency, the alternating components of the signal vanish and only constant components remain. The following equations show the generalized I_i and Q_i component of a frequency w_i.

$$V_{i,I} = \frac{A_i A_{\text{ref}}}{2} \cdot \cos(\varphi_i) = I_i \tag{8}$$

$$V_{i,Q} = \frac{A_i A_{\text{ref}}}{2} \cdot \sin(\varphi_i) = Q_i \tag{9}$$

3. Results of Resistive/Capacitive Test Unit

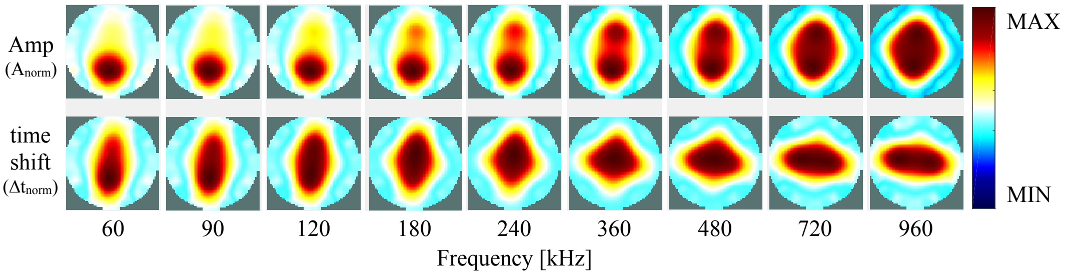

Figure S1. Time-difference images of the test unit using the inhomogeneities at the positions shown in Figure 12 for both amplitude and time shift of measurements at different frequencies. For each frequency, the image was reconstructed separately and autoscaled individually, so that also the smallest differences can be visualized. The MAX and MIN in the color-scale are correspondent to the maximum and minimum value of each figure.

4. Results of Self-Experiment

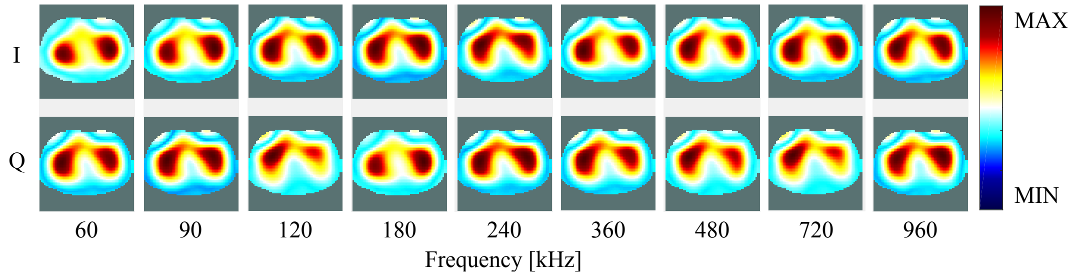

(a)

Figure S2. *Cont.*

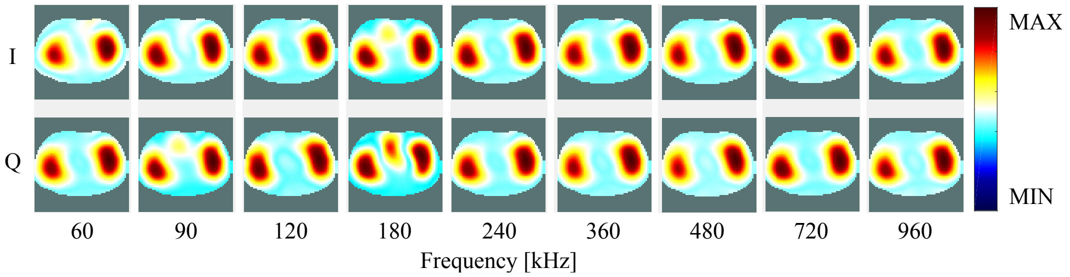

(b)

Figure S2. Functional images for the real (I) and imaginary (Q) components of the measured signals at the P1 (**a**) and P2 (**b**) positions of the electrode belt at diverse frequencies. For each frequency, the image was reconstructed separately and autoscaled individually, so that also the smallest differences can be visualized. The MAX and MIN in the color-scale are correspondent to the maximum and minimum value of each figure.

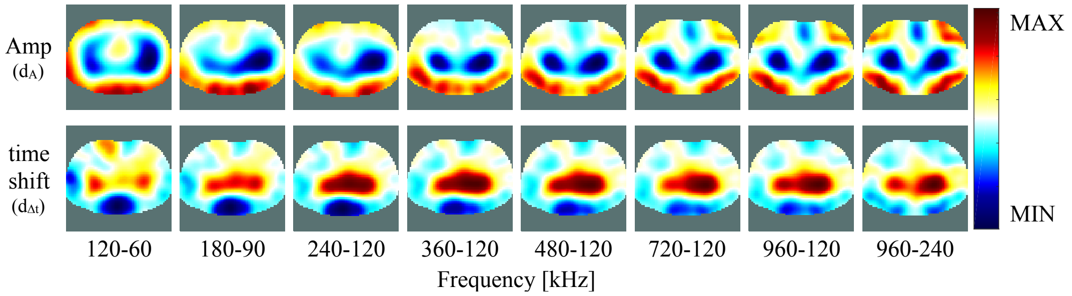

(a)

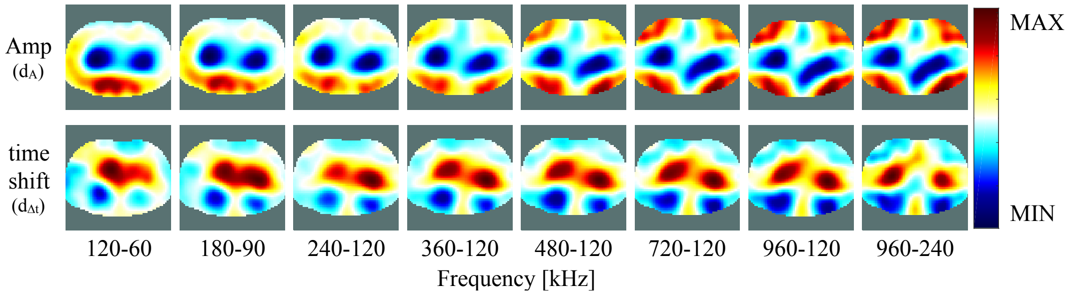

(b)

Figure S3. Frequency-difference images of the P1 (**a**) and P2 (**b**) positions of the electrode belt. For each frequency, the image was reconstructed separately and autoscaled individually, so that also the smallest differences can be visualized. The MAX and MIN in the color-scale are correspondent to the maximum and minimum value of each figure.
